# Evenamide, as an add-on to antipsychotics, benefits patients with treatment resistant schizophrenia: 6-month interim results from the first 100 patients in an ongoing international randomized study

**DOI:** 10.1192/j.eurpsy.2023.1329

**Published:** 2023-07-19

**Authors:** R. Anand, R. Hartman, V. Lucini, R. Giuliani, A. Turolla, G. Chinellato

**Affiliations:** 1 R&D department, Anand Pharma Consulting AG, St. Moritz, Switzerland; 2 NeurWrite LLC, Morristown, United States; 3 R&D department, Newron Pharmaceuticals SpA, Bresso, Italy

## Abstract

**Introduction:**

Treatment resistance schizophrenia (TRS) develops in ~ 30% of patients in about 5 years from starting treatment with 5-HT2/D2 APs, resulting in increased morbidity, suicidality, and mortality. Findings from neurochemistry, neurometabolism, functional imaging in TRS patients indicate abnormalities in glutamatergic neurotransmission (Moghaddam B et al 2012; 37 4-15) rather than excess of dopamine synthesis (Demjaha A et al 2014; 75 11-3; Mouchlianitis E et al 2016; 42 744-52), suggesting the need to add a drug that attenuates glutamate release. Evenamide, a selective inhibitor of voltage-gated Na^+^ channels, is devoid of biological activity at >130 CNS targets, normalizes glutamate release without affecting basal levels, and demonstrated benefits in animal models of psychosis as monotherapy and as an add on to APs (including clozapine), reversing deficits produced by amphetamine, scopolamine, phencyclidine, or ketamine

**Objectives:**

Studies 014/015 were designed to evaluate the safety and preliminary efficacy of evenamide given orally at 3 fixed doses (7.5, 15 and 30 mg bid) in patients with TRS not responding to a therapeutic dose of an AP. Assessment of efficacy was based on changes of PANSS and CGI-S/C, while tolerability was assessed based on all safety measures

**Methods:**

Study 014 is a 6-week, randomized, rater-blinded, international study with completers continuing assigned doses for an additional 46 weeks in an extension study (Study 015). Patients were initially randomized to 7.5 or 15 mg bid; the Independent Safety Monitoring Board (ISMB) allowed randomization to 30 mg bid after reviewing safety data from the first 50 patients. At baseline, patients were moderately to severely ill (CGI-S of 4 to 6), with a PANSS total score of 70-90 and predominant positive symptoms (score of 4 or more on at least 2 core symptoms and a PANSS positive total score ≥ 20), along with functional deficits (GAF ≤50). Efficacy ratings were performed by a psychiatrist blinded to the evenamide dose. Data were analyzed as a single group using descriptive statistics to assess changes from baseline to endpoint (Week 30)

**Results:**

Interim, group-blinded, 30-week results for safety and efficacy data (PANSS and CGI) for the first 100 patients (including 6 on 30 mg bid) will be presented. Patients randomized to 7.5, 15, and 30 mg bid had all safety and efficacy data pooled in a single group to maintain the blind in the study. All results will be submitted to the ISMB, relevant health authorities and the FDA

**Conclusions:**

This trial is the first international TRS trial of an NCE AP used as an add-on to a single typical or atypical AP. Results of this study may change the treatment of future TRS patients

**Disclosure of Interest:**

None Declared

